# Adjudicated myocarditis and multisystem illness trajectory in healthcare workers post-COVID-19

**DOI:** 10.1136/openhrt-2022-002192

**Published:** 2023-02-23

**Authors:** Robert Sykes, Andrew J Morrow, Alex McConnachie, Anna Kamdar, C Bagot, Hannah Bayes, Kevin G Blyth, Michael Briscoe, Heeraj Bulluck, David Carrick, Colin Church, David Corcoran, C Delles, Iain Findlay, Vivienne B Gibson, Lynsey Gillespie, Douglas Grieve, Pauline Hall Barrientos, Antonia Ho, N N Lang, David J Lowe, Vera Lennie, Peter MacFarlane, Kaithlin J Mayne, Patrick Mark, Alasdair McIntosh, Ross McGeoch, Christopher McGinley, Connor Mckee, Sabrina Nordin, Alexander Payne, Alastair Rankin, Keith E Robertson, Nicola Ryan, Giles H Roditi, Naveed Sattar, David B Stobo, Sarah Allwood-Spiers, Rhian Touyz, Gruschen Veldtman, Sarah Weeden, Stuart Watkins, Paul Welsh, Ryan Wereski, Kenneth Mangion, Colin Berry

**Affiliations:** 1 School of Cardiovascular and Metabolic Health, University of Glasgow, Glasgow, UK; 2 Cardiology, Golden Jubilee National Hospital West of Scotland Regional Heart and Lung Centre, Glasgow, UK; 3 Cardiology, Queen Elizabeth University Hospital, Glasgow, UK; 4 Robertson Centre for Biostatistics, University of Glasgow, Glasgow, UK; 5 Haematology, Glasgow Royal Infirmary, Glasgow, UK; 6 Respiratory Medicine, Glasgow Royal Infirmary, Glasgow, UK; 7 School of Cancer Sciences, University of Glasgow, Glasgow, UK; 8 Respiratory Medicine, Queen Elizabeth University Hospital, Glasgow, UK; 9 Cardiology, Leeds General Infirmary, Leeds, West Yorkshire, UK; 10 Cardiology, University Hospital Hairmyres, East Kilbride, South Lanarkshire, UK; 11 Scottish Pulmonary Vascular Unit, Golden Jubilee Hospital, Clydebank, UK; 12 Cardiology, Royal Alexandra Hospital, Paisley, Renfrewshire, UK; 13 Haemostasis and Thrombosis, Glasgow Royal Infirmary, Glasgow, UK; 14 Project Management Unit, Glasgow Clinical Research Facility, Glasgow, UK; 15 Respiratory Medicine, Royal Alexandra Hospital, Paisley, Renfrewshire, UK; 16 Medical Physics, NHS Greater Glasgow and Clyde, Glasgow, Glasgow, UK; 17 MRC-University of Glasgow Centre for Virus Research, School of Infection and Immunity, University of Glasgow, Glasgow, UK; 18 Emergency Medicine, Queen Elizabeth University Hospital, Glasgow, UK; 19 Cardiology, Aberdeen Royal Infirmary, Aberdeen, Aberdeen, UK; 20 Cardiology, University Hospital Crosshouse, Kilmarnock, East Ayrshire, UK; 21 Radiology, NHS Greater Glasgow and Clyde, Glasgow, Glasgow, UK; 22 Centre for Cardiovascular Science, The University of Edinburgh, Edinburgh, UK

**Keywords:** COVID-19, Magnetic Resonance Imaging, Myocarditis, Risk Factors

## Abstract

**Background:**

We investigated the associations of healthcare worker status with multisystem illness trajectory in hospitalised post-COVID-19 individuals.

**Methods and results:**

One hundred and sixty-eight patients were evaluated 28–60 days after the last episode of hospital care. Thirty-six (21%) were healthcare workers. Compared with non-healthcare workers, healthcare workers were of similar age (51.3 (8.7) years vs 55.0 (12.4) years; p=0.09) more often women (26 (72%) vs 48 (38%); p<0.01) and had lower 10-year cardiovascular risk (%) (8.1 (7.9) vs 15.0 (11.5); p<0.01) and Coronavirus Clinical Characterisation Consortium in-hospital mortality risk (7.3 (10.2) vs 12.7 (9.8); p<0.01). Healthcare worker status associated with less acute inflammation (peak C reactive protein 48 mg/L (IQR: 14–165) vs 112 mg/L (52–181)), milder illness reflected by WHO clinical severity score distribution (p=0.04) and shorter duration of admission (4 days (IQR: 2–6) vs 6 days (3–12)).

In adjusted multivariate logistic regression analysis, healthcare worker status associated with a binary classification (probable/very likely vs not present/unlikely) of adjudicated myocarditis (OR: 2.99; 95% CI (1.01 to 8.89) by 28–60 days postdischarge).

After a mean (SD, range) duration of follow-up after hospital discharge of 450 (88) days (range 290, 627 days), fewer healthcare workers died or were rehospitalised (1 (3%) vs 22 (17%); p=0.038) and secondary care referrals for post-COVID-19 syndrome were common (42%) and similar to non-healthcare workers (38%; p=0.934).

**Conclusion:**

Healthcare worker status was independently associated with the likelihood of adjudicated myocarditis, despite better antecedent health. Two in five healthcare workers had a secondary care referral for post-COVID-19 syndrome.

**Trial registration number:**

NCT04403607.

**WHAT IS ALREADY KNOWN ON THIS TOPIC**

- The protection of healthcare workers is essential to the provision of health services during an infectious disease pandemic.
- Occupational exposure is a risk factor for infection from communicable disease including COVID-19.
- Few prospective studies of the trajectory of COVID-19 disease in healthcare workers have been undertaken, and the burden of severe illness on workers with high level of exposure requires investigation.

**WHAT THIS STUDY ADDS**

- We undertook a prospective study of 168 patients including 36 healthcare workers hospitalised due to COVID-19, performing cardiovascular and renal MRI at 28–60 days, with contemporary CT pulmonary and coronary angiography, and CT Thorax. We also obtained blood and urine biomarkers and participants completed patient-reported outcome measure questionnaires.
- Despite better antecedent health than non-healthcare workers, adjudicated myocarditis was more likely in healthcare workers.
- Post-COVID-19 syndrome was common but no greater than nonhealthcare workers.

**HOW THIS STUDY MIGHT AFFECT RESEARCH, PRACTICE OR POLICY**

- In this prospective cohort, occupational exposure to COVID-19 in healthcare workers associated with myocarditis and two in five healthcare workers required secondary care referrals for postCOVID-19 syndrome.
- Infection control precautions and the provision of appropriate personal protective equipment to reduce exposure to COVID-19 are required in professions with greater exposure

## Introduction

Symptoms of post-COVID-19 are common, leading to increased demands on healthcare services.^1–8^ Protecting healthcare workers from occupational health problems, notably nosocomial communicable disease such as COVID-19,^9^ is crucial. Healthcare workers are at an increased risk of infection due to frequent and prolonged exposure to COVID-19 from aerosols or contaminated secretions in clinical areas.^10–13^ Few prospective studies of COVID-19 disease in healthcare workers have been undertaken.

We hypothesised that healthcare workers might be at increased risk of severe infection and disease complications following hospitalisation with COVID-19 due to occupational exposure compared with non-healthcare workers. We investigated this hypothesis using multisystem imaging, biomarkers and their changes over the short and medium term. Patient-reported outcome measures recorded health status and physical and psychological function, and electronic health records were used to establish clinical outcomes and healthcare use.

## Methods

### Design

The Chief Scientist Office Cardiovascular and Pulmonary Imaging in SARS Coronavirus disease-19 (CISCO-19) study involved a prospective, observational, multicentre, longitudinal, secondary care cohort study design to assess the trajectory of multiorgan injury in survivors of COVID-19 during convalescence.^14 15^ Participants were assessed at enrolment (visit 1) and again, 28–60 days following discharge from hospital (visit 2). At each visit, clinical information, a 12-lead digital ECG, blood and urine biomarkers and patient-reported outcome measures were acquired. Cardiorenal MRI followed by contemporary chest CT, including pulmonary and coronary angiography, were acquired at the second visit. An analysis based on self-reported healthcare worker status was prespecified.

### Participant identification

CISCO-19 was performed in three hospitals in the West of Scotland (population 2.2 million). Surviving patients receiving hospital care for COVID-19, with or without admission, were prospectively screened using an electronic healthcare information system (TrakCare, InterSystems, USA) and reports identifying PCR-positive hospital inpatients with COVID-19 (Roche Cobas 6800 or Seegene SARS-CoV-2 PCR).

### Eligibility criteria

The inclusion criteria were: (1) age ≥18 years old; (2) history of an unscheduled attendance to hospital secondary to COVID-19 with positive COVID-19 PCR result; (3) ability to comply with study procedures and (4) ability to provide written informed consent. Imaging results were reported according to contemporary national guidelines by accredited radiologists.^16^

The exclusion criteria were: (1) contraindication to MRI or (2) lack of informed consent.

### Screening

A screening log was prospectively completed and recorded reasons for being ineligible.

### Diagnosis of myocardial injury

Myocardial injury was defined according to the Fourth Universal Definition of Myocardial Infarction. High sensitivity troponin I (Abbott Architect STAT TnI assay) was measured in hospitalised patients with sex-specific upper reference limit >99th percentile: men >34 ng/L, women >16 ng/L.

### Multimodality imaging

#### CT

Comprehensive pulmonary assessment was performed by acquisition of an initial low radiation dose helical scan of the thorax. Cardiopulmonary transit times were assessed by a contrast bolus timing scan. Non-contrast followed by contrast-enhanced angiographic breath-hold ECG-gated volumes were acquired and timed for optimum pulmonary and systemic arterial (coronary) opacification. Non-contrast acquisitions were obtained in patients with severe renal dysfunction precluding contrast administration.

To assess for the presence and extent of flow-limiting coronary artery disease and coronary calcification, CT coronary angiography was performed incorporating fractional flow reserve CT assessment (FFR_CT_; HeartFlow, Redwood City, California). Obstructive coronary artery disease was defined by an FFR_CT_ ≤0.80 in the presence of a corresponding coronary lesion, taking the lowest value in the vessel. Pulmonary vascular imaging was performed to assess for pulmonary arterial thrombus (embolism).^17^ Pulmonary features associated with COVID infection, for example, atelectasis, reticulation and/or architectural distortion, ground-glass opacity and pre-existing lung damage, for example, emphysema were delineated by CT. Incidental findings including cardiac and extracardiac were reported and managed according to local standards of care.

#### Cardiovascular MRI

Patients were invited to undergo protocol-directed MRI in the convalescent phase, 28–60 days after discharge. MRI was acquired using a research-dedicated 3.0 Tesla (3T) scanner (MAGNETOM Prisma, Siemens Healthineers, Erlangen, Germany) with two 18-channel surface coils placed anteriorly and a 32-channel spine coil placed posteriorly. The scan protocol included cine-imaging of cardiac anatomy and function and myocardial tissue characterisation using multiparametric techniques, namely, myocardial native longitudinal relaxation time (T1, milliseconds) before and following intravenous gadolinium contrast media (Magnevist, Bayer Healthcare), mapping transverse relaxation time (T2 in milliseconds), first pass contrast-enhanced perfusion and late gadolinium enhancement imaging.

The modified Lake Louise criteria were used to diagnose definite myocardial inflammation (abnormal T2 and T1 (native T1, late gadolinium enhancement or extracellular volume)) or probable myocardial inflammation (abnormal: T2 or T1).^18 19^ UK Biobank reference ranges were used to interpret cardiac structure and function,^20^ and scanner-specific contemporary local reference ranges defined thresholds for localised abnormalities in myocardial T1-relaxation and T2-relaxation times. Patients with severe renal dysfunction were not excluded and underwent MRI with or without contrast media according to the site radiology protocol.

#### Renal MRI

Multiparametric renal MRI included anatomical imaging and tissue characterisation by measurement of native T1 and T2 relaxation times (ms). Corticomedullary differentiation reflects variance in tissue contrast on T1-weighted imaging due to a shorter cortical T1 relaxation time relative to the medulla reflecting differences in water content between these tissues.^21 22^ Kidney disease may diminish corticomedullary differentiation, reported here as a ratio of T1 cortex divided by T1 medulla.^21 22^

### Blinding

Patients completed health status questionnaires prior to imaging and their scan results. Core analyses were performed by researchers independent of patient characteristics, control status or other results. The cardiologists who formed the clinical adjudication panel were unaware of the patient-reported outcome measures. They were also unaware of the adjudications made by the other panel members.

### Outcomes

#### Primary outcome

The predefined primary outcome was a diagnosis of adjudicated myocarditis (myocardial inflammation), a subgroup of acute myocardial injury. Adjudication was undertaken by a panel of cardiologists independent of the research team.

Myocarditis was clinically suspected in the presence of at least one clinical finding and at least one diagnostic test criterion, in the absence of (1) angiographically detectable, flow-limiting coronary artery disease (coronary stenosis ≥50%, FFR_CT_<0.80); (2) alternative extracardiac causes or known pre-existing cardiovascular disease which could explain the syndrome (eg, valve disease, congenital heart disease, hyperthyroidism, etc). The likelihood of myocarditis increases with each criterion met. In asymptomatic patients, two or more diagnostic criteria were required.

### Adjudication of the primary outcome

We prespecified an adjudication procedure for the primary outcome to reduce ascertainment bias, involving a panel of cardiologists with specialty accreditation. The reviews were undertaken according to a prespecified charter.

Fourteen independent consultant cardiologists were provided with information on the European Society of Cardiology Working Group on Myocardial and Pericardial Disease position statement on myocarditis,^18^ a charter, and training cases, including a vignette with clinical presentation, severity of illness and objective findings from clinical and research procedures. Cases were pseudo-anonymised and assessed by at least five cardiologists in turn. The likelihood or not of myocarditis was scored using an ordinal Likert system (not present/unlikely/probable/very likely). A weighted summative score would inform the categorisation of the likelihood of myocarditis using clinical and diagnostic criteria in line with clinical guidelines. The median likelihood determined the final diagnosis for each case and control. Each rater reassessed 30 cases to assess intraobserve variability and test–retest reliability. Categorisations were also dichotomised into not present/unlikely versus probable/very likely myocarditis to produce a binary classification (no vs yes).

### Health status and patient-reported outcome measures

Questionnaires were completed at enrolment (visit 1) and 28–60 days after discharge from the hospital. The generic (EuroQOL EQ-5D-5L questionnaire and the Brief Illness Perception Questionnaire assessed self-reported health status of the participants.^23 24^ An assessment for depression and anxiety was performed using the Patient Health Questionnaire-4.^25^ The Duke Activity Status Index predicted maximal oxygen utilisation (mL/kg/min) and functional capacity. A higher score reflected higher degrees of physical function.^26^ The International Physical Activity Questionnaire—Short Form (IPAQ-SF) measures physical activity, intensity and time spent sitting down. The score from IPAQ-SF reflected the total physical activity at each visit per participant in metabolic equivalent minutes per week.^27^

### Longitudinal follow-up for clinical outcomes

Clinical research team members assessed electronic health records without participant contact in line with the protocol and a predefined charter for follow-up assessments of serious adverse events (SAEs), including death and rehospitalisation, andNational Health Service (NHS) resource utilisation, including procedures, outpatient clinic visits and medication prescriptions. Cardiovascular and respiratory SAE were independently reviewed and adjudicated by the clinical event committee. The events were entered into the database coordinated by the clinical trials unit.

### Statistics

The statistical analyses, including a predefined analysis of healthcare worker status, were described in a statistical analysis plan. The statistical methods are described in the tables.

### Sample size calculation

To detect an association between a history of pre-existing cardiovascular disease and incident myocardial inflammation (myocarditis) determined based on median likelihood from the clinical adjudication committee, we assumed the presence of prior cardiovascular disease in 25% of the study population and that the incidence of myocardial inflammation in those with and without prior cardiovascular disease would be 33% and 10%, respectively.^28^ Thirty-five patients with prior cardiovascular disease and 105 without would provide 80% power to detect this difference. It was envisaged that 10%–15% of the participants might have incomplete data, for example, artefact or claustrophobia, and, therefore, a target sample size of 160 would be recruited to complete the imaging visit.

Associations between healthcare worker status adjudicated the likelihood of myocarditis and mechanistic biomarkers, patient-reported outcome measures and the primary and secondary outcomes were assessed. Missing data are reported. CIs accompany significance tests with two-sided p values for estimated effect sizes and measures of association without adjustment for multiplicity. The p values for subgroup differences were calculated using the Fisher Exact test and the Kruskal-Wallis test for categorical and continuous data. A p value of less than 0.05 was considered statistically significant.

### Trial management and timelines

The study was conducted in line with the current *Guidelines for Good Clinical Practice in Clinical Trials* and *Strengthening the Reporting of Observational Studies in Epidemiology* guidelines^29^ and coordinated by a Study Management Group. A Scientific Steering Group had oversight of the study. The CISCO-19 study was initially considered by the NHS Glasgow Patient and Public Involvement group during 2020. The study was also considered by lay members of the research ethics committee. Updates from the study have been contributed to the long COVID-19 Scotland group meetings, which have taken place approximately quarterly since 2020.

### Sources of funding

This was an investigator-initiated clinical study funded by the Chief Scientist Office of the Scottish Government (COV/GLA/Portfolio project number 311300). The funder had no role in the design, conduct (non-voting TSC member), data analysis and interpretation, manuscript writing or dissemination of the results. CB, CD, NS, RT were supported by the British Heart Foundation (RE/18/6/34217).

The MRI study involved technologies provided by Siemens Healthcare and the National Institutes of Health. HeartFlow (HeartFlow, Redwood City, California) provided FFR_CT_. The study was cosponsored by NHS Greater Glasgow & Clyde Health Board and the University of Glasgow.

## Results

**Figure 1:**
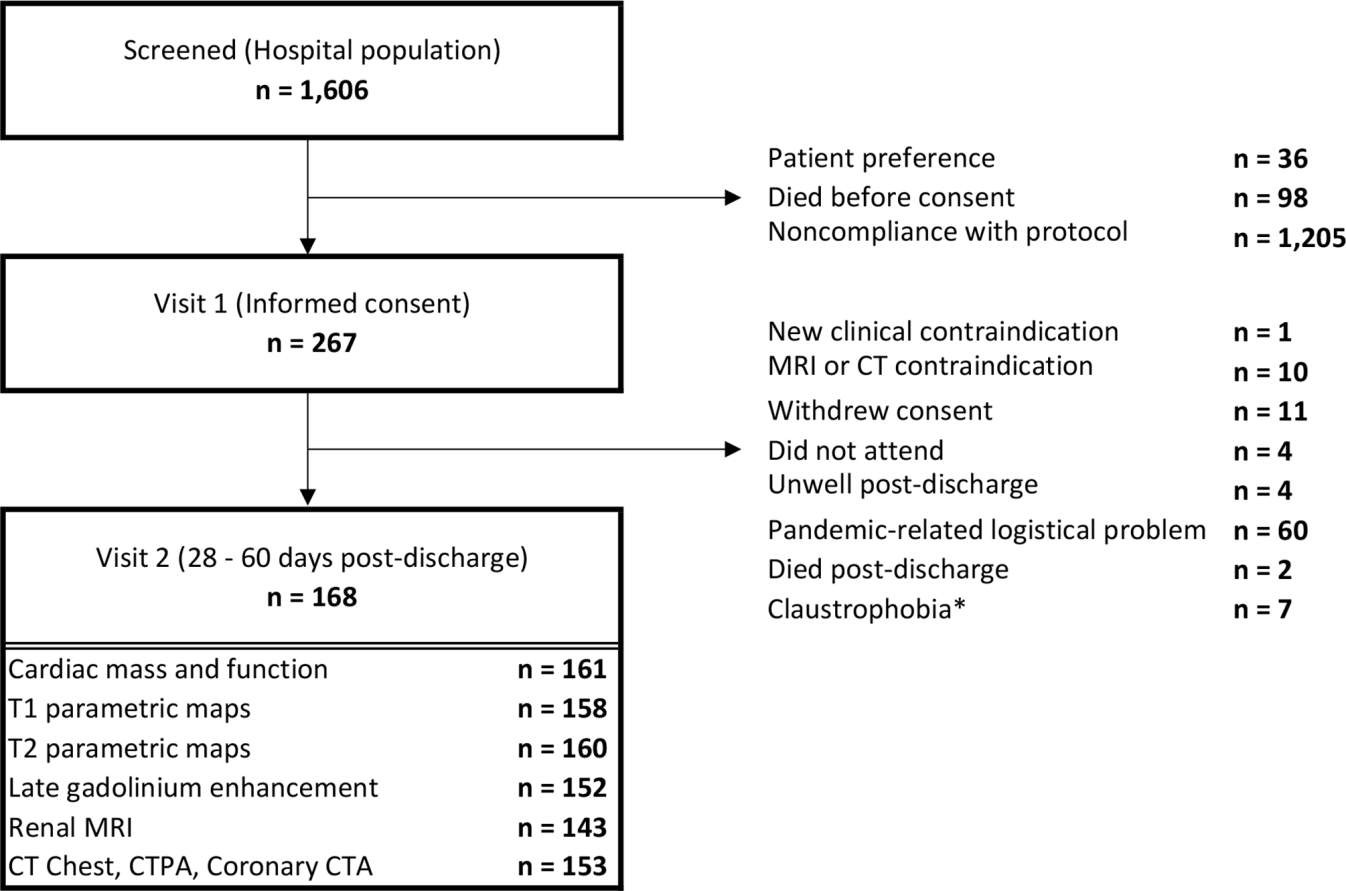
Flow diagram of the clinical study. The procedures involved screening hospitalised patients with COVID-19, defined by a PCR-positive result for SARS-CoV-2 in a nasopharyngeal swab and obtaining written informed consent. A PCR-positive result defines the analysis population. Serial investigations were initiated in-hospital or early post-discharge (visit 1) and then repeated in association with multiorgan imaging at 28–60 days post-discharge (visit 2). Clinical follow-up continued for on average (SD) 450 (88) days (range 290, 627 days) post-discharge. *Claustrophobia prevented the completion of the full imaging protocol in 14 patients—however, 7 provided data for biomarkers, patient-reported outcome measures and limited imaging acquisitions before abandonment.

One thousand six hundred and six patients who received hospital care for COVID-19 were screened between 22 May 2020 and 16 March 2021, and 267 patients provided written informed consent(figure 1).

One hundred and sixty-eight patients, including 36 (21%) healthcare workers, were evaluated at 28–60 days after the last episode of hospital care, of whom 154 completed cardiovascular MRI with stress-perfusion, renal MRI and cross-sectional CT coronary and pulmonary angiography with high-resolution CT of the thorax. The remaining patients partially completed the imaging due to renal impairment precluding contrast and severe breathlessness, preventing adenosine stress-perfusion as protocolled or abandoned due to body habitus or claustrophobia. Blood biomarkers and questionnaires were obtained from these individuals, and, therefore, they were included as intention-to-treat. The average age was 54 years, 88% were white, 44% were women, 46% had a history of cardiovascular disease or treatment, 41% were in the lowest quintile of social deprivation and 21% were healthcare workers table 1.

**Table 1:** Clinical characteristics of the study population by healthcare worker status.

	Covid-19	Covid-19		
	All	Healthcare worker	Non-healthcare worker	P value*
	n=168	n (%) = 36 (21)	n (%) = 132 (79)	HCW vs non-HCW
Demographic				
Age±SD, years	54.2±11.8	51.3 (8.7)	55.0 (12.4)	0.09
Male sex, n (%)	94 (56%)	10 (28%)	84 (64%)	<0.001
Female sex, n (%)	74 (44%)	26 (72%)	48 (36%)	0.168
Most deprived SIMD quintile (Q1), n (%)	65 (41%)	17 (52%)	48 (38%)	0.168
Ethnicity, n (%)				
White	147 (88%)	28 (78%)	119 (90%)	0.051
Asian	15 (9%)	7 (19%)	8 (6%)	
Other	6 (4%)	1 (3%)	5 (4%)	
Presenting characteristics, mean (SD)				
Body mass index, kg/m^2^	30.9 (7.2)	28.8 (7.5)	31.4 (7.1)	0.053
Heart rate, bpm	95 (19)	92 (20)	96 (19)	0.209
Systolic blood pressure, mmHg	130 (20)	127 (18)	130 (21)	0.43
Peripheral oxygen saturation, %	93 (6)	95 (5)	93 (7)	0.072
WHO clinical severity score for COVID-19, n (%)				
Hospitalised, no oxygen therapy	54 (32%)	17 (47%)	37 (28%)	0.044
Oxygen therapy by mask or nasal prongs	77 (46%)	12 (33%)	65 (49%)	
Non-invasive ventilation	22 (13%)	2 (6%)	20 (15%)	
Mechanical ventilation	15 (9%)	5 (14%)	10 (8%)	
Radiology, chest radiograph or CT scan, n (%)				
Typical features of COVID-19	118 (76%)	23 (70%)	95 (77%)	0.399
Normal	24 (15%)	8 (24%)	16 (13%)	
Acute COVID-19 therapy, n (%)				
Oxygen	114 (68%)	19 (53%)	95 (72%)	0.043
Steroid	91 (54%)	16 (44%)	75 (57%)	0.194
Antiviral	43 (26%)	6 (17%)	37 (28%)	0.2
Non-invasive respiratory support	33 (20%)	5 (14%)	28 (21%)	0.478
Intensive care	24 (14%)	6 (17%)	18 (14%)	0.601
Invasive ventilation	15 (9%)	5 (14%)	9 (7%)	0.182
Cardiovascular history, n (%)				
Smoking: never	110 (65%)	28 (78%)	82 (62%)	0.08
Smoking: former	47 (28%)	5 (14%)	42 (32%)	
Smoking: current	11 (7%)	3 (8%)	8 (6%)	
Hypercholesterolemia	80 (48%)	12 (33%)	68 (52%)	0.061
Hypertension	56 (33%)	8 (22%)	48 (36%)	0.162
Diabetes mellitus	37 (22%)	6 (17%)	31 (23%)	0.498
Chronic kidney disease	7 (4%)	1 (3%)	6 (5%)	1
Cardiovascular disease and/or treatment	77 (46%)	14 (39%)	63 (48%)	0.451
Risk scores, mean (SD)				
ISARIC-4C in-hospital mortality risk, %	11.5 (10.1)	7.3 (10.2)	12.7 (9.8)	0.004
Q-Risk 3, 10 year cardiovascular risk, %	13.7 (11.2)	8.1 (7.9)	15.0 (11.5)	0.006
Charlson Comorbidity Index	1.8 (1.8)	1.4 (1.6)	1.9 (1.8)	0.132
Laboratory results, index admission				
Initial haemoglobin, mean (SD), g/L	141 (16)	138 (13)	142 (16)	0.276
Initial platelet count, mean (SD), x10^9^/L	238 (92)	234 (78)	239 (95)	0.778
Initial white cell count, mean (SD), x10^9^/L	7.4 (5.4)	5.9 (1.9)	7.8 (6.0)	0.073
Peak D-Dimer, mean (SD), ng/mL	1609 (5342)	1409 (2239)	1658 (5865)	0.853
Acute kidney injury, n (%)	20 (15%)	4 (13%)	16 (15%)	1
Peak hs-troponin I, median (IQR), ng/L	4.0 (3.0, 11.0)	4.0 (2.0, 14.8)	4.0 (3.0, 11.0)	0.237
Peak ferritin, mean (SD), mg/L	356 (168, 906)	243 (88, 610)	368 (189, 947)	0.142
Peak C reactive protein, median (IQR), mg/L	104 (37, 180)	48 (14, 165)	112 (52, 181)	0.028
HbA1c, mean mmol/mol Hb, %	47.9 (18.1)	45.6 (16.1)	48.4 (18.5)	0.472
Timelines				
Duration of admission, median (IQR), days	5 (2, 11)	4 (2, 6)	6 (3, 12)	0.0496
Symptom onset to enrolment, median (IQR), days	26 (13, 38)	29 (15, 38)	24 (13, 38)	0.38
Hospital discharge to visit 2 (28–60 days post-discharge), median (IQR), days	48 (37, 55)	42 (35, 51)	48 (38, 56)	0.11

Missing data in healthcare worker post-COVID-19 patients: postcode for SIMD, n=3; typicality of radiology for COVID-19, n=3; standard care blood tests: D-Dimer, n=16; HbA1c, n=9; ferritin, n=6; troponin I, n=8. Missing data in non-healthcare worker post-COVID-19 patients: postcode for SIMD, n=5; typicality of radiology for COVID-19, n=9; standard care blood tests: D-Dimer, n=50; HbA1c, n=13; ferritin, n=11; troponin I, n=11.GFR—glomerular filtration rate was estimated using the Chronic Kidney Disease Epidemiology equation,^33^ In the control group, the Abbott Architect CMIA SARS-CoV-2 IgG assay was used to confirm the absence of prior infection with COVID-19. The primary outcome evaluation (visit 2) was scheduled for 28–60 days post-discharge.

*Categorical data are summarised as frequency and percentage and compared between groups using Fisher’s Exact tests. Continuous data are summarised as mean and SD, or median and interquartile range (IQR, defined as the upper and lower quartiles), and compared between groups using Kruskal-Wallis tests. A p value of less than 0.05 was considered significant.

HbA1c, haemoglobin A1c; HCW, Healthcare worker; ISARIC-4C, Coronavirus Clinical Characterisation Consortium; SIMD, Scottish Index of Multiple Deprivation.

Two (1.2%) patients had received a single dose of a SARS-CoV-2 vaccine prior to hospitalisation. Regarding COVID-19 therapy, 68% received oxygen, 54% received steroids, 26% received antiviral drug therapy, 20% received non-invasive respiratory support and 8% received invasive ventilation.

### Healthcare workers

Compared with non-healthcare workers, healthcare workers were of comparable age (51.3 (8.7) years versus 55.0 (12.4) years; p=0.09) and were more often women (26 (72%) vs 48 (38%); p<0.01). They had a lower 10-year percentage cardiovascular risk (%) (8.1 (7.9) vs 15.0 (11.5); p<0.01) and a lower ISARIC-4C in-hospital mortality risk (7.3 (10.2) vs 12.7 (9.8); p<0.01). Healthcare worker status was associated with less severe acute inflammation (peak C reactive protein (CRP) 48 mg/L (IQR: 14 to 165) vs 112 mg/L (52 to 181), illness severity reflected by the WHO clinical severity score distribution (p=0.04) and shorter duration of admission (4 days (IQR: 2 to 6) vs 6 days (3 to 12)).

### Multisystem phenotyping and adjudicated myocarditis

#### Electrocardiology

Electrocardiographic features of myocarditis criteria defined by contemporary criteria did not differ by healthcare worker status (table 2).

**Table 2:** Multisystem phenotyping by healthcare worker status: serial electrocardiography, biomarkers of inflammation, metabolism, renal function, haemostasis, and heart, lung, and kidney imaging at 28–60 days post-discharge

	COVID-19 All	Healthcare worker	Non-healthcare worker	
	n=168	n=36	n=132	P value*
ECG, n (%)				
Admission (n=160)				
**Myopericarditis criteria**	34 (21%)	8 (24%)	26 (21%)	0.814
Enrolment (n=157)				
**Myopericarditis criteria**	50 (32%)	7 (22%)	43 (34%)	0.206
28–60 days post-discharge (n=150)				
**Myopericarditis criteria**	37 (25%)	9 (28%)	28 (24%)	0.646
CT chest 28–60 days post-discharge				
Ground glass opacity and/or consolidation, n (%)	67 (44%)	10 (29%)	57 (48%)	0.053
Reticulation and/or architectural distortion, n (%)	44 (29%)	7 (20%)	37 (31%)	0.287
Pulmonary arterial thrombus, n (%)	6 (4%)	2 (6%)	4 (3%)	0.616
**Visual estimate of % of total lung area abnormal, mean (SD**)	14.2 (19.2)	**7.9** (**14.1**)	**16.0** (**20.1**)	**0.028**
CT coronary angiogram 28–60 days post-discharge				
Obstructive coronary artery disease, n (%)	19 (13%)	2 (6%)	17 (15%)	0.246
FFR_CT_, patient-level (all coronary arteries)				
Median FFR_CT_, mean (SD)	0.93 (0.03)	**0.94** (**0.02**)	**0.93 (0.03**)	**0.037**
Minimum FFR_CT_≤0.80, n (%)	49 (38%)	9 (30%)	40 (40%)	0.392
Cardiovascular MRI 28–60 days post-discharge				
LV ejection fraction, mean (SD), %	54.2 (9.7)	55.8 (7.5)	53.7 (10.2)	0.258
**LV mass index, mean (SD), g/m^2^**	91.7 (25.4)	**79.4** (**24.1**)	**95.5** (**24.7**)	**<0.001**
RV ejection fraction, mean (SD), %	50.9 (10.6)	50.9 (10.0)	50.8 (10.8)	0.987
Myocardial tissue characterisation				
**Increased global T1 (>1233 ms), n (%**)	**54** (**35%**)	**18** (**51%**)	**36** (**31%**)	**0.028**
Increased global T2 (>44 ms), n (%)	10 (7%)	4 (11%)	6 (5%)	0.238
**Increased global extracellular volume (>27.4%), n (%**)	**71** (**51%**)	**22** (**69%**)	**49** (**46%**)	**0.027**
Late gadolinium enhancement				
Myocardial late gadolinium enhancement, n (%)	30 (20%)	6 (17%)	24 (20%)	0.811
Ischaemic distribution, n (%)	8 (6%)	2 (6%)	6 (6%)	1.000
Non-ischaemic distribution, n (%)	24 (17%)	5 (15%)	19 (17%)	0.799
Myocardial inflammation (Lake Louise criteria), n (%)				
No evidence (0/2)	15 (10%)	0 (0%)	15 (13%)	0.054
Probable (1/2)	71 (46%)	17 (49%)	54 (46%)	
Definite (2/2)	67 (44%)	18 (51%)	49 (42%)	
Renal MRI, mean (SD)				
**Average volume of right and left kidneys, mL**	**153** (**32**)	**142** (**29**)	**156** (**32**)	**0.024**
Average cortex T1 of right and left kidneys, ms	1542 (61)	1537 (72)	1543 (59)	0.699
Average medulla T1 of right and left kidneys, ms	1933 (68)	1932 (84)	1933 (66)	0.930
Biomarkers at enrolment, central laboratory				
C reactive protein, median (IQR), mg/L	5.1 (1.5, 20.8)	4.0 (0.6, 18.3)	5.4 (1.6, 22.2)	0.235
High sensitivity troponin I, median (IQR), ng/L	3 (2, 6)	3 (2, 4)	3 (2, 6)	0.138
NT proBNP, median (IQR), pg/mL	108 (57, 258)	80 (46, 178)	118 (60, 283)	0.092
**Ferritin, median (IQR**), **µg/L**	**341 (190, 667**)	**221 (123, 403**)	**404 (214, 686**)	**0.018**
Total cholesterol, mean (SD), mmol/L	4.9 (1.4)	**5.2** (**1.3**)	4.8 (1.4)	0.089
**HDL cholesterol, mean (SD), mmol/L**	**1.1** (**0.4**)	**1.2** (**0.4**)	**1.0** (**0.3**)	**0.028**
**ST2, median (IQR**), **ng/mL**	**30.6 (18.8, 55.9**)	**21.0 (14.2, 37.3**)	**34.0 (20.9, 66.6**)	**0.001**
eGFR, median (IQR), mL/min/1.73 m^2^	95.7 (85.0, 105.1)	96.6 (83.7, 106.1)	95.1 (85.6, 104.9)	0.812
**Von Willebrand Factor: GP1bR, mean (SD**)	**240** (**130**)	**200** (**105**)	**251** (**134**)	**0.038**
Von Willebrand Factor: Ag, mean (SD)	245 (150)	202 (92)	257 (160)	0.053
Biomarkers at 28–60 days post-discharge, central laboratory				
C reactive protein, mean (SD), mg/L	1.9 (0.9, 3.6)	1.5 (0.6, 3.6)	1.9 (1.2, 3.6)	0.282
**High sensitivity troponin I, median (IQR**), **ng/L**	2 (1, 4)	**2 (1, 2**)	**2 (2, 4**)	**0.017**
NT proBNP, median (IQR), pg/mL	83 (54, 187)	108 (53, 186)	82 (56, 186)	0.667
Ferritin, median (IQR), ug/L	144 (68, 255)	100 (53, 219)	147 (75, 289)	0.056
Total cholesterol, mean (SD), mmol/L	4.6 (1.2)	4.7 (0.9)	4.5 (1.2)	0.375
**Triglycerides, mean (SD), mmol/L**	**2.0** (**1.1**)	**1.7** (**0.8**)	**2.1** (**1.2**)	**0.041**
**HDL cholesterol, mean (SD), mmol/L**	**1.1** (**0.3**)	**1.2** (**0.4**)	**1.0** (**0.3**)	**0.047**
**Endothelin-1, median (IQR**), **pg/ml,**	**2.3 (1.9, 2.9**)	**2.0 (1.7, 2.6**)	**2.3 (2.0, 3.0**)	**0.029**
**IL-6, median (IQR**), **pg/mL**	**2.7 (2.0, 4.5**)	**2.30 (1.5, 3.7**)	**3.0 (2.0, 4.6**)	**0.035**
ST2, median (IQR), ng/mL	19.9 (14.4, 26.8)	16.5 (13.3, 21.6)	21.0 (14.8, 28.6)	0.058
**Direct bilirubin, median (IQR**), **µmol/L**	**1.5 (0.8, 2.1**)	**0.8 (0.8, 1.6**)	**1.6 (0.8, 2.2**)	**0.038**
**Thrombin clotting time, mean (SD), s**	**12.7** (**1.3**)	**12.4** (**1.3**)	**12.8** (**1.2**)	**0.046**
Fibrinogen, mean (SD), g/L	3.4 (1.4)	3.8 (1.9)	3.3 (1.2)	0.081
Factor VIII, mean (SD), IU/dL	150 (63)	154 (55)	149 (66)	0.692
Antithrombin, mean (SD), IU/dL	109 (17)	110 (15)	108 (18)	0.542
**Protein S, mean (SD**)	**99.5** (**20.7**)	**91.1** (**19.0**)	**101.8** (**20.7**)	**0.008**
Protein C, mean (SD)	111.6 (23.8)	110.4 (20.6)	111.9 (24.7)	0.755
**Von Willebrand Factor: GP1bR, mean (SD**)	144 (79)	134 (50)	146 (85)	0.429
Von Willebrand Factor: Ag, mean (SD)	165 (95)	169 (95)	164 (95)	0.808
*Urine biomarkers*				
Albumin: creatinine ratio, mean (SD), enrolment	3.1 (7.9)	2.5 (4.5)	3.3 (8.7)	0.604
Albumin: creatinine ratio, mean (SD), 28–60 days post-discharge	4.0 (10.9)	1.4 (2.3)	4.7 (12.2)	0.112

Missing data in healthcare workers and non-healthcare workers post-COVID-19 patients (admission, enrolment, 28–60 days)—ECG myopericarditis criteria—n=2, n=4, n=4; n=6, n=7, n=14; Missing data in healthcare workers post-COVID-19 patients at 28–60 days and non-healthcare workers—CT chest atelectasis, reticulation, ground glass—n=1, n=13; pulmonary arterial thrombus—n=3, n=17; CT coronary angiogram 28–60 days healthcare workers and non-healthcare workers: Agatston score—n=1, n=18; FFR_CT_—n=6, n=33; Cardiovascular MRI 28–60 days post-discharge: left ventricular end-diastolic volume index, left ventricular end-systolic volume index, left ventricular ejection fraction, left ventricular strain—n=0, n=16; left ventricular mass – n=0, n=16; right ventricular end-diastolic volume index, right ventricular systolic volume index, n=0, n=18; right ventricular ejection fraction, n=0, n=17; global T1—n=1, n=14; global T2—n=1, n=14; global extracellular volume—n=4, n=25; late gadolinium enhancement—n=1, n=14; ischaemic distribution—n=3, n=24; non-ischaemic distribution—n=2, n=23; myocardial inflammation—n=1, n=14. Missing data in core laboratory blood biomarkers, healthcare workers (enrolment; 28–60 days) and non-healthcare workers (enrolment; 28–60 days)—eGFR—n=2, n=6; n=2, n=12; C reactive protein—n=0, n=8; n=1, n=9; high sensitivity troponin I—n=0, n=10; n=1, n-11; ΝΤ-proBNP—n=0, n=10; n=1, n=14; total cholesterol, triglycerides, HDL cholesterol—n=0, n=8; n=1, n=8; ICAM-1, CVAM-1, Endothelin-1, IL-6, ST2, p=selectin—n=2, n=5; n=2, n=12; LDH, Haptoglobin, bilirubin—n=2, n=7; n=2, n=13; Fibrinogen—n=1, n=3; n=2 n=12; D-Dimer—n=1, n=3; n=2, n=11; Factor VIII—n=1, n=3; n=2, n=11; Antithrombin—n=1, n=3; n=2, n=12; Protein C—n=1, n=3; n=2, n=12; Protein S—n=2, n=3; n=3, n=12; VWF:GP1bR—n=1, n=3; n=2, n=11; VWF:Ag—n=1, n=3; n=2, n=11.

*Categorical data are summarised as frequency and percentage and compared between groups using Fisher's Exact tests. Continuous data are summarised as mean and standard deviation, or median and interquartile range (IQR, defined as the upper and lower quartiles), and compared between groups using Kruskal-Wallis tests. There were no differences for healthcare workers versus non-healthcare workers in premature atrial contraction, premature ventricular contraction, atrial fibrillation or flutter, left and right ventricular strain, (%), pericardial thickening, n (%), pericardial effusion, n (%), right atrial area, mean (SD), cm^2^, left atrial area, mean (SD), cm^2^ at enrolment or during follow-up. A p value of less than 0.05 was considered significant.

aPTT, activated partial thromboplastin time; ECV, extracellular volume; EDV, end-diastolic volume; EF, ejection fraction; eGFR (CKD-EPI), estimated glomerular filtration rate using the Chronic Kidney Disease Epidemiology; ESV, end-systolic volume; FFR_CT_, fractional flow reserve computed tomography; HbA1c, haemoglobin A1c; HDL, high density lipoprotein; LV, left ventricle; MESA, multi-ethnic study of atherosclerosis; NT-proBNP, N-terminal pro B-type natriuretic peptide; PT, prothrombin time; RV, right ventricle; T1, longitudinal relaxation time; T2, transverse relaxation time; TCT, thrombin clotting time; vWF:Ag, von Willebrand factor antigen.

#### CT chest, coronary and pulmonary angiography

At 28–60 days following discharge from the hospital, healthcare worker status was associated with less abnormal lung volume (healthcare workers: 7.9% (14.1) visual estimate of total lung volume vs non-healthcare workers 16.0% (20.1)) compared with non-healthcare workers. Median CT fractional flow reserve was greater in healthcare workers, reflecting a lower burden of coronary artery disease (table 2).

#### Cardiovascular MRI

Compared with non-healthcare workers, healthcare workers had increased myocardial native T1 relaxation times and extracellular volume, consistent with myocardial inflammation (table 2). Myocardial mass was lower in healthcare workers reflecting the higher proportion of women in the healthcare worker group.

#### Renal MRI

There was no difference in renal inflammation between healthcare workers and non-healthcare workers.

### Primary outcome

Healthcare worker status was associated with a binary classification (probable/very likely vs not present/unlikely) of adjudicated myocarditis during adjusted multivariate logistic regression analysis (OR: 2.99; 95% CI (1.01 to 8.89)) (table 3).

**Table 3:** Univariate and multivariable associates of adjudicated myocarditis (primary outcome), including demographic characteristics

	Univariate		Multivariable	
	OR (95% CI)	P value	OR (95% CI)	P value
Demographics				
Age (per 10 years)	0.87 (0.67 to 1.14)	0.304	1.02 (0.72 to 1.45)	0.897
**Sex (female vs male**)	**2.01 (1.06 to 3.82**)	**0.033**	1.45 (0.64 to 3.26)	0.372
Ethnicity (Other vs White)	2.17 (0.79 to 5.98)	0.133		
SIMD (Quintile 2 vs Most Deprived)	0.49 (0.20 to 1.21)	0.120		
SIMD (Quintile 3 vs Most Deprived)	0.41 (0.14 to 1.21)	0.108		
SIMD (Quintile 4 vs Most Deprived)	0.58 (0.20 to 1.70)	0.319		
SIMD (Quintile 5 vs Most Deprived)	1.10 (0.43 to 2.81)	0.838		
**Healthcare worker (yes vs no**)	**2.31 (1.05 to 5.10**)	**0.038**	**2.99 (1.01 to 8.89**)	**0.048**
Body Mass Index (per 5 kg/m^2^)	1.11 (0.89 to 1.39)	0.364		

ORs, 95% CIs and p values derived from logistic regression models. A p value of less than 0.05 was considered significant. Univariate models include one predictor only. The multivariable model was adjusted for age and sex and included any other factors found to have p<0.05 in univariate analysis (ie, healthcare worker status, acute kidney injury and HbA1c). The OR relates to the specified between-group difference (categorical predictors) or increase (continuous predictors).

EQ-5D-5L, EuroQol Research Foundation EQ-5D five level instrument; SIMD, Scottish Index of Multiple Deprivation.

The total variance across all adjudication ratings was 0.885 and between adjudicated ratings was 0.725 using an ordinal scale of values from 1 to 4 for the likelihood of myocarditis. The between-subject variation to the total variation ratio was 0.82. Adjudicating cardiologists each repeated 30 cases in a blinded fashion to assess intraobserver reliability. The average-weighted kappa statistic for classifying the likelihood of myocarditis into four levels was 0.69, and for the binary classification (probable/very likely vs not present/unlikely), it was 0.79.

### Health status

Compared with non-healthcare workers, at enrolment and 28–60 days postdischarge, healthcare workers had a better health-related quality of life, lower illness perception, lower levels of anxiety and depression, higher levels of vigorous physical activity and similar predicted maximal oxygen utilisation (mL/kg/min) reflecting aerobic exercise capacity (table 4).

**Table 4:** Patient-reported outcome measures of health status, illness perception, anxiety and depression and physical function by healthcare worker status

Enrolment	All COVID-19 n=163	Healthcare worker		P value
		Yes n=36	No n=127	
Visit 2 (28–60 days post-discharge from hospital)	n=167	n=36	n=131	
Health status, mean (SD)				
Health-related quality of life EQ-5D-5L score at enrollment	0.74 (0.21)	0.75 (0.18)	0.73 (0.22)	0.758
Health-related quality of life EQ-5D-5L score 28–60 days post-discharge	0.77 (0.23)	0.81 (0.16)	0.75 (0.25)	0.171
Patient assessed EQ-5D-5L score at enrolment, EQ-5D-5L score	61.3 (21.7)	59.9 (20.4)	61.7 (22.1)	0.656
Patient assessed EQ-5D-5L score at 28–60 days post-discharge,	72.9 (19.5)	74.9 (17.5)	72.3 (20.1)	0.478
Illness perception, mean (SD)				
Brief Illness Perception Questionnaire score at enrollment	42.2 (12.6)	40.2 (11.8)	42.8 (12.8)	0.270
Brief Illness Perception Questionnaire score 28–60 days post-discharge	36.4 (14.8)	34.7 (13.9)	36.8 (15.1)	0.442
Anxiety and depression, mean (SD)				
PHQ-4 anxiety score at enrollment	2.12 (2.12)	1.97 (2.01)	2.17 (2.16)	0.634
**PHQ-4 anxiety score at 28–60 days post-discharge**	**1.80** (**2.02**)	**1.19** (**1.65**)	**1.97** (**2.09**)	**0.042**
PHQ-4 depression score at enrollment	2.16 (1.92)	1.71 (1.74)	2.28 (1.95)	0.120
PHQ-4 depression score at 28–60 days post-discharge	1.81 (1.93)	1.28 (1.61)	1.96 (1.99)	0.060
PHQ-4 total score at enrollment	4.28 (3.76)	3.69 (3.47)	4.45 (3.83)	0.289
**PHQ-4 total score at 28–60 days post-discharge**	**3.61** (**3.73**)	**2.47** (**3.03**)	**3.93** (**3.86**)	**0.038**
Physical function				
IPAQ category at enrollment, n (%)				
High	11 (7%)	4 (12%)	7 (6%)	0.372
Moderate	17 (11%)	4 (12%)	13 (11%)	
Low	121 (81%)	24 (75%)	97 (83%)	
IPAQ category at 28–60 days post-discharge, n (%)				
High	24 (17%)	4 (14%)	20 (18%)	0.846
Moderate	43 (31%)	10 (36%)	33 (30%)	
Low	72 (52%)	14 (50%)	58 (52%)	
Duke Activity Status Index at enrolment	19.0 (17.8)	18.9 (19.3)	19.0 (17.4)	0.985
Duke Activity Status Index at 28–60 days post-discharge	23.9 (17.5)	25.0 (15.7)	23.6 (18.1)	0.669
Predicted maximal O_2_ utilisation (mL/kg/min) at enrollment	17.8 (7.6)	17.7 (8.3)	17.8 (7.5)	0.985
Predicted maximal O_2_ utilisation (mL/kg/min) at 28–60 days postdischarge	19.9 (7.5)	20.4 (6.7)	19.8 (7.8)	0.669

Categorical data are summarised as frequency and percentage and compared between groups using Fisher’s Exact tests. Continuous data are summarised as mean and SD and compared between groups using Kruskal-Wallis tests. A p value of less than 0.05 was considered significant.

EQ-5D-5L, EuroQol Research Foundation EQ-5D five level instrument; IPAQ, International Physical Activity Questionnaire; PHQ-4, Patient Health Questionnaire-4.

### Serious adverse events

Follow-up was continued to 13 December 2021, for all participants. The mean (SD, range) duration of follow-up after hospital discharge was 450 (88) days (range 290–627 days).

Four patients died, including two deaths before and two after visit 2 at 28–60 days following hospital discharge after COVID-19 (table 5). No deaths occurred among healthcare workers. Twenty-two (17%) patients died or were rehospitalised, including one healthcare worker (p=0.038). One hundred and thirteen (67.9%) patients with post-COVID-19 had an episode of outpatient secondary care, including 27 (75%) healthcare workers and 86 (65%) non-healthcare workers. Referrals for symptoms consistent with NICE188 guideline criteria for long COVID-19 were very common in both groups but not significantly different by healthcare worker status (15 (42%) vs 50 (38%); p=0.934).

**Table 5:** Clinical outcomes by healthcare worker status

	All COVID-19 n=168	Healthcare worker		P value
		Yes n=36	No n=132	
Duration of follow-up				
Days to visit 3 or death, (median IQR)	420 (370, 446)	425 (368, 555)	420 (380, 442)	0.326
*Outcomes,* n (%)				
**Death or hospitalisation (any cause**)	**23** (**14%**)	**1** (**3%**)	**22** (**17%**)	**0.038**
Death (any cause)	2 (1%)	0 (0%)	2 (2%)	0.457
Cardiovascular death	1 (1%)	0 (0%)	1 (1%)	0.597
Renal death	0 (0%)	0 (0%)	0 (0%)	–
Respiratory death	0 (0%)	0 (0%)	0 (0%)	–
**Hospitalisation (any cause**)	**23** (**14%**)	**1** (**3%**)	**22** (**17%**)	**0.038**
Secondary care (outpatients)				
Any outpatient care	113 (67%)	27 (75%)	86 (65%)	0.192
Acute COVID-19 (<28 days)	15 (9%)	4 (11%)	11 (8%)	0.614
Ongoing COVID-19 (28–84 days)	22 (13%)	4 (11%)	18 (14%)	0.702
Long COVID-19 (>84 days)	65 (39%)	15 (42%)	50 (38%)	0.934

For clinical outcomes, data are the number and percentage of participants with at least one event during follow-up. P values are from log-rank tests of the time to the first event. A p value of less than 0.05 was considered significant.

## Discussion

We investigated multisystem pathology, patient-reported health status, aerobic capacity and clinical outcomes for 14 months after hospitalisation for COVID-19. One in seven patients died or was readmitted to the hospital, and two-thirds had an episode of outpatient secondary care. Post-COVID-19 syndrome was prevalent, with 65 (39%) of all patients referred to secondary care with NICE188 guideline criteria for long COVID-19.

Almost one-quarter of the patients were healthcare workers and they were mostly women. Despite less severe acute illness and better antecedent health, compared with non-healthcare workers, healthcare workers had a threefold higher likelihood of adjudicated myocarditis, reflecting deep organ involvement of COVID-19. The aetiology of myocardial inflammation may be direct viral myocarditis or myocardial inflammation reflecting multisystem illness. Reverse causality may be relevant in that individuals with reasonably good background health have a greater reserve to withstand COVID-19 such that in those individuals who eventually become sufficiently unwell to require hospital care, the severity of COVID-19 is more pronounced. Healthcare workers have enhanced occupational exposure to SARS-CoV-2 in their workplace, as evidenced by the exposure of the clinicians in the research team, all of whom developed COVID-19 during the study, despite adhering to recommendations for personal protective equipment and social distancing measures. Several of the non-clinical research staff also developed COVID-19. There have been previous concerns regarding more significant viral load and increased exposure to aerosolised viral particles in healthcare workers.^30^ This may explain why healthcare workers had evidence of systemic involvement in deep organs, that is, myocarditis, despite seemingly lower levels of systemic inflammation reflected by CRP.

Despite better antecent health and less severe COVID-19 illness initially, post-COVID-19 syndrome was just as common in healthcare workers as non-healthcare workers. Referrals to secondary care for symptoms persisting beyond 84 days in keeping with long COVID-19 were made in two in five patients, and the proportions were similar between both groups. The overall burden of post-COVID-19 symptoms was high. The participants were enrolled before the roll-out of the COVID-19 vaccination programme in the United Kingdom, and all but two patients (1%) were unvaccinated. Occupational exposure and the risk of persistent symptoms have important implications for the safety and well-being of healthcare staff and health service workforce planning in the health service. Since vaccination prevents COVID-19 and reduces the likelihood of long COVID-19 symptoms,^31^ our results highlight the importance of healthcare workers being vaccinated against SARS-CoV-2.

A strength of this study is the blinded adjudication process which included a panel of at least five cardiologists to assess the likelihood of myocarditis in each case. A core laboratory approach blinded researchers to occupational status, demographics, disease severity and outcomes and preserved objectivity during the analysis of study imaging, electrocardiograms and blood or urine biomarkers. Statistical analysis was undertaken independently from the research team by two biostatisticians. There was no missing data for follow-up assessments, which were completed using electronic health records.

### Limitations

To minimise COVID-19 transmission to our healthcare staff, imaging was scheduled from 28 days postdischarge. This approach aligns with the International Severe Acute Respiratory and Emerging Infection Coronavirus Clinical Characterisation Consortium study.^32^ Since acute imaging was not performed, some pathologies may have resolved by 28 days. Selection and ascertainment bias was minimised but not eliminated.

### Conclusions

The illness trajectory of COVID-19 in healthcare workers involves a greater degree of myocardial involvement despite fewer cardiovascular risk factors and comorbidities. The burden of post-COVID-19 syndrome was high, affecting 42% of healthcare workers in this study, with implications for workforce planning. Preventive therapy for post-COVID-19 syndromes and longer term studies of prognosis are warranted.

## Data Availability

Data are available upon reasonable request. Data requests will be considered by the Steering Group, which includes representatives of the Sponsor, the University of Glasgow, senior investigators independent of the research team, and the chief investigator. The Steering Group will take account of the scientific rationale, ethics, logistics, and resource implications. Data access requests should be submitted by email to the Chief Investigator (Colin Berry, corresponding author). The source data include the deidentified numerical data used for the statistical analyses and deidentified imaging scans (MRI, CT) and ECGs. Data access will be provided through the secure analytical platform of the Robertson Centre for Biostatistics. This secure platform enables access to deidentified data for analytical purposes without the possibility of removing the data from the server. The Steering Group will consider requests for the transfer of deidentified data (including source imaging scans); if approved, a collaboration agreement would be expected. The Steering Group will consider any cost implications, and cost recovery would be expected on a not-for-profit basis.
